# *De novo* dedifferentiated SDH-deficient gastrointestinal stromal tumor with MDM2 amplification: case report and literature review

**DOI:** 10.3389/fonc.2023.1233561

**Published:** 2023-09-14

**Authors:** Qi-Xing Gong, Ying Ding, Wei-Ming Zhang, Jia-Wen Zhang, Zhi-Hong Zhang

**Affiliations:** Department of Pathology, The First Affiliated Hospital of Nanjing Medical University, Nanjing, China

**Keywords:** gastrointestinal stromal tumor, dedifferentiation, SDH, MDM2, p53

## Abstract

The dedifferentiation of the gastrointestinal stromal tumors (GISTs) has been reported in a small number of cases, usually under the pressure of the tyrosine kinase inhibitor (TKI) treatment. Herein, we described a *de novo* dedifferentiated GIST with the SDH deficiency in a 32-year-old Chinese woman. The tumor was located on the lesser curvature of the gastric antrum, measuring 4.1x9.1 cm^2^. Microscopically, the tumor was composed of 2 distinct morphological populations, mild epithelioid cells arranged in the multinodular growth pattern and hyperchromatic spindle cells arranged in the fascicular or sheet-like architecture. The two zones showed different immunophenotypes. The former proved to be an epithelioid GIST with the positive expression for C-KIT, DOG-1, and CD34, and the latter expressed the CKpan and P53, but negative for the C-KIT, DOG-1, and CD34. However, the SDHB staining was negative in both areas. Genetically, the next-generation sequencing (NGS) analysis showed the SDHC mutation (p.S48*) in both components and the MDM2 amplification was only in the spindle cell area. The lesion was diagnosed as the SDH-deficient GIST with the epithelial cell dedifferentiation. We proposed that the P53 associated gene alteration or other alternative escape mechanisms for the KIT-independent signaling pathways might play a role in the dedifferentiation.

## Introduction

Gastrointestinal stromal tumors (GISTs) are the most common primary mesenchymal neoplasms of the gastrointestinal tract. They are categorized into different subtypes based on the different genetic mutations and various clinicopathological characteristics (1). Approximately 85% of the GISTs harbor active mutations of the KIT or PDGFRA oncogene. Among the remaining wildtype GISTs, succinate dehydrogenase (SDH)-deficient GISTs are the most frequently found neoplasms, accounting for about 5-10% of the overall occurrence. The SDH-deficient GISTs have a predilection for children and young adult populations and have gastric involvement, multinodular growth pattern, epithelioid cytology, lymphovascular invasion, and a greater risk of metastasis to the regional lymph nodes or liver when compared to the classical GISTs. Immunophenotypically, most GISTs, including SDH-deficient GISTs, show strong and diffuse expression of KIT (CD117) and DOG1. However, in an extremely rare circumstance, tumor cells with morphologically high-grade progression, such as undifferentiated pleomorphic sarcoma (2), or heterologous epithelial (3), myogenic (4), or angiosarcomatous differentiation (3), could lose the GIST immunohistochemical characteristics. This was described as dedifferentiated GISTs, which can occur under the pressure of the tyrosine kinase inhibitor (TKI) treatment or develop *de novo*. The previous findings suggested that the genetic instability, such as loss of heterozygosity (LOH) or low-level KIT amplification, might have correlations with the dedifferentiation after therapy. On the other hand, alternative escape mechanisms for the KIT-independent signaling pathway might play a role in the *de novo* dedifferentiation (5). However, more cases are needed to investigate the underlying mechanisms. Herein, we described a case of SDH-deficient GIST with the heterologous epithelial dedifferentiation that occurred *de novo* with additional MDM2 gene amplification in the dedifferentiated area, which has not been reported previously.

## Case presentation

### Clinical summary

**Figure 1:**
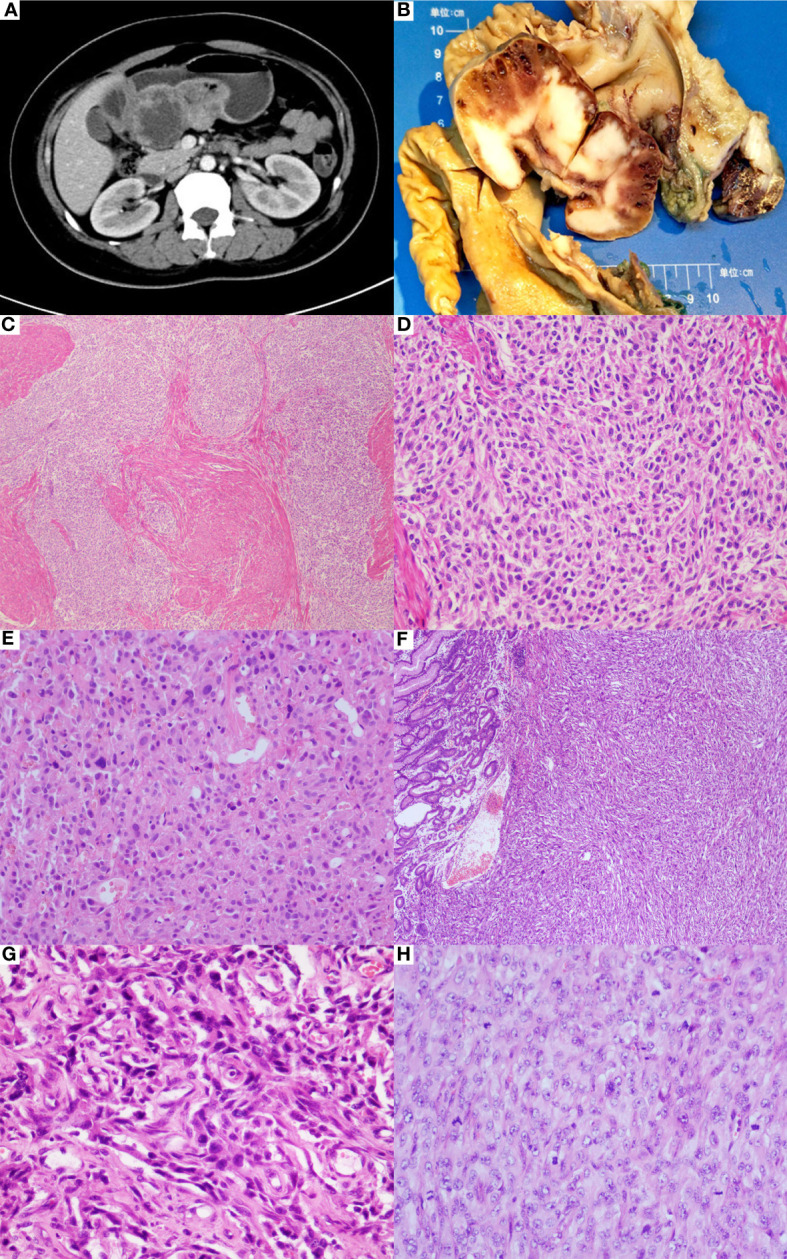
Clinicopathological features of gastric differentiated GIST. **(A)** CT examination revealed a mass located on the lesser curvature of gastric antrum. **(B)** The gastrectomy specimen showed a gray-white mass with hemorrhage and small cystic changes in the cut surface. **(C)** Microscopically, somewhere tumor cells were predominantly epithelioid cells arranged in multinodular pattern (HE*40). **(D)** The epithelioid cells were mild with variably eosinophilic cytoplasm, reminiscent of SDH-deficient GIST(HE*200). **(E)** There were some moderate to severe atypical cells spotted in the mild epithelioid cell groups (HE*200). **(F)** Conversely, sheet of anaplastic spindle cells was present elsewhere, similar to the pattern seen in the biopsy specimen (HE*40). **(G)** The anaplastic cells showed highly pleomorphic nuclear with brisk mitoses, accompanied with proliferation of mesenchymal fibroblasts (HE*400). **(H)** Undifferentiated small round cells were noticed (HE*400).

The patient was a 32-year-old Chinese woman complaining of progressive dizziness and fatigue for the last two years. Initial laboratory tests showed a low hemoglobin level (32 g/dl, normal: 105–135 g/dl), and therefore the patient was diagnosed with an iron deficiency anemia. The CT examination revealed a mass located on the lesser curvature of the gastric antrum, measuring 4.1x9.1 cm^2^ (**Figure 1A**). The lesion showed uneven enhancement after gadolinium-based contrast administration, indicating interior necrosis. In addition, multiple enlarged lymph nodes were noticed in the small omental sac, with the largest one measuring 3.1x2.1 cm^2^ by the contrast-enhancement. 345

eet proliferating atypical spindle cells with brisk mitoses. Immunohistochemically, the tumor cells expressed CKpan, but the cells were negative for CD34, CD117, DOG1, LCA, Syn, CD31, EGR, and S100. The proliferation index of the ki67 was 75%. The initial pathological diagnosis was a malignant tumor with poor differentiation, but it was recommended for a surgical biopsy evaluation to clarify the specific tumor type. The patient underwent radical distal gastrectomy and 6 courses of postoperative chemotherapy. The available follow-up period was about 42 months, and the evaluation showed no clinical or radiological signs of local recurrence or metastasis.

### Pathological findings

The gastrectomy specimen showed a 10x8x4 cm^3^ mass in the gastric antrum, bulging into the cavity with facial ulceration. The main body of the lesion was situated in the submucosa and muscularis propria, focally invading gastric serosa. The cut surface was grayish-white in appearance with the hemorrhage and small cystic changes (**Figure 1B**). Multiple enlarged lymph nodes were surrounding the stomach.

**Figure 2:**
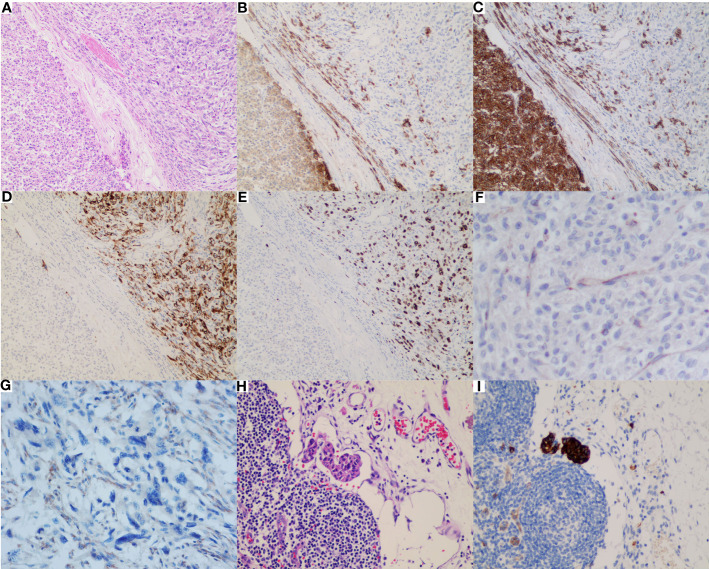
Immunophenotype and clinical behavior of the differentiated SDH-deficient GIST. **(A)** The two zones were abruptly transitioned (HE*200). The mild epithelioid cells showed a positive reaction for CD117 **(B)**, DOG-1 **(C)** and a negative reaction for CKpan **(D)** and P53 **(E)**, while the analpastic spindle cells were on the contrary. However, both components lost the expression of SDHB (FG) while the internal controls, such as the background vascular endothelial cells **(F)** and non-neoplastic fibroblasts **(G)**, were positively stained. Metastatic epithelioid cells **(H)** expressing C-KIT **(I)** were observed in the gastric omental lymph nodes.

Microscopically, the tumor was composed of 2 distinct morphological populations. In some areas, tumor cells were predominantly epithelioid cells in a multinodular pattern, involving the gastric wall (**Figure 1C**). On the high power of microscopic evaluation, the cells were relatively uniform with variably eosinophilic cytoplasm and mild to moderate atypical nuclei, and the mitotic count was around 4 per 50 HPFs (**Figure 1D**). However, some moderate to severe atypical cells were sparsely noticed (**Figure 1E**). Another part of the area was dominated by the spindle cells, similar to the pattern seen in the biopsy specimen (**Figure 1F**). The spindle cells showed a fascicular or sheet-like architecture, with ill-defined cell borders, variably amphophilic or palely eosinophilic cytoplasm, highly pleomorphic nucleus, dense chromatin, and brisk mitoses (about 75 per 50 HPFs) (**Figure 1G**). Multinucleated giant tumor cells and undifferentiated small round cells were frequently noticed (**Figure 1H**). The two zones were abruptly transitioned and showed a different immunophenotype (**Figures 2A–E**). The mild epithelioid cells showed a positive reaction for the C-KIT, DOG-1, and CD34, and a negative reaction for smooth muscle actin (SMA), desmin, S100, CD31, ERG, CKpan, and P53. Contrastingly, highly pleomorphic spindle cells were positive for the CKpan and P53, but negative for the C-KIT, DOG-1, and CD34, SMA, desmin, S100, CD31, and ERG. The average Ki67 proliferation index of the epithelioid cells and the spindle cells were 2% and 50%, respectively. However, the staining for the SDH-B was negative in both areas (**Figures 2F, G**).

Based on these findings, the tumor was considered as SDH-deficient GIST with epithelial dedifferentiation. A total of 32 lymph nodes were examined, and two of them revealed the metastatic epithelioid cells (**Figure 2H**) expressing C-KIT (**Figure 2I**), DOG-1, and CD34, which corresponded to the conventional SDH-deficient GIST.

**Figure 3:**
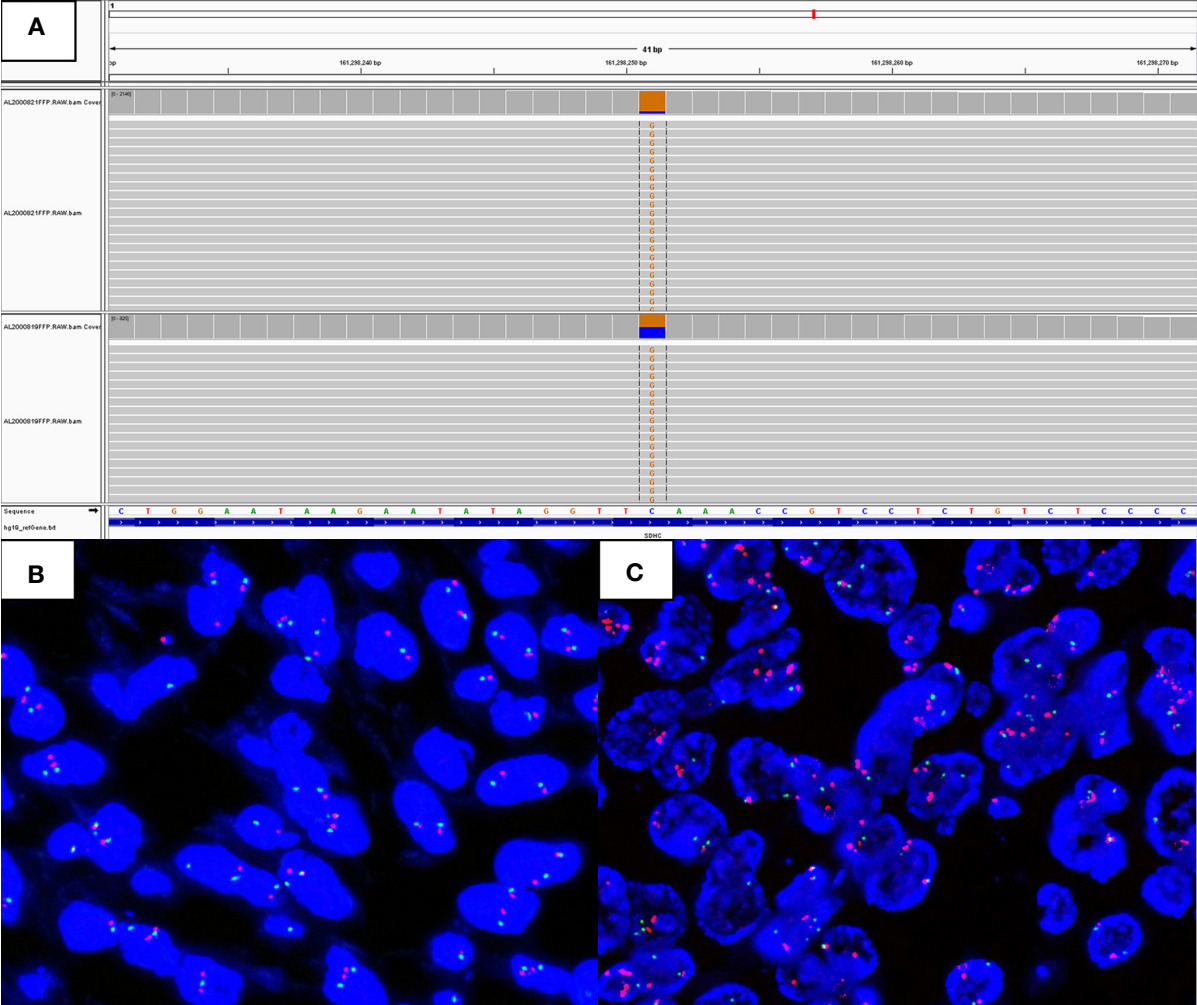
Molecular features of the differentiated SDH-deficient GIST. The classical area and the differentiated area presented with SDHC truncation mutation **(A)**. FISH assays verified MDM2 amplification in the dedifferentiated region **(C)**, compared with the classic region **(B)**.

Furthermore, the NGS analysis was done on the formalin-fixed paraffin-embedded tissues from both areas. Although there was no difference in the KIT or PDGFRA genotype between the two zones, the SDHC mutation (p.S48*) terminating the SDHC protein translation prematurely, was identified from both zones (**Figure 3A**). Additionally, the MDM2 and CARD11 amplifications, and missense mutation of SOX10 (p.P366S), RHOA (p.R70S), and FANCA (p.Q1235H) were explored in the dedifferentiated area. Using a commercially available Vysis LSI MDM2 dual-color probe (Abbott Laboratories), the FISH assays were performed on 3-µm-thick sections from the FFPE tumor tissue blocks, confirming that the copy number of the MDM2 signal increased in the dedifferentiated region (**Figures 3B, C**).

## Discussion

Dedifferentiation, a cellular process reverting the cells to a less differentiated stage, has been described in various neoplasms, including soft tissue tumors such as well-differentiated liposarcoma, solitary fibrous tumor, chondrosarcoma, osteosarcoma, and chordoma. The term “dedifferentiation” or “transdifferentiation” was initially used for the GISTs to define three cases of morphological and phenotypical transition from the conventional KIT-positive GISTs to the high-grade KIT-negative neoplasms following the imatinib (IM) treatment (6). To date, at least 26 cases of dedifferentiation GIST have been reported in the currently published literature (2–14). We summarize its clinicopathological characteristics in **Supplementary Table 1** as follows.

There were 20 males and 6 females, with a wide age distribution at the diagnosis, ranging from 18-75 years (median, 53 years old). Seventeen cases occurred as tumor progression after a short or long time (ranging from 3 m to 8 y) treatment with tyrosine kinase inhibitor (TKI). Most of them presented as metastatic or recurrent (involving the liver, peritoneum, mesentery, and omentum) after surgical removal of the primary tumor (in the stomach, duodenum, intestine, and rectum) and the TKI treatment. Few cases were diagnosed by biopsy and experienced progression as nodules within a mass or regrowth of a preexisting tumor under the TKI therapy. The *de novo* differentiated GISTs are extremely rare, with only 9 cases reported, including ours. Among them, 5 cases occurred in the stomach, 1 in the small intestine, 1 in the rectum, and 2 in the metastatic locations, which had the metastatic implants at the diagnosis.

Histologically, two components, the well-differentiated GISTs, and the anaplastic/dedifferentiated components were observed synchronously or heterochronously. Normally, the dedifferentiated component loses the usual morphological features of the GISTs, whereas they appear in various histological patterns, presenting with heterogenous phenotype, and accompanied by highly cellular, marked pleomorphism, and frequent mitoses and necrosis. Among the available data from the reported cases, 10 morphologically exhibited rhabdomyoblastic differentiation, mimicking embryonal or pleomorphic rhabdomyosarcoma (4), with the aberrant expression of desmin, myoD1, and myogenin, although the original GIST was completely negative for them. Nine dedifferentiated GISTs exhibited epithelioid or epithelial differentiation, with one showing large round and epithelioid cells, one demonstrating tubular papillary growth pattern, and seven expressing cytokeratin. Three cases were illustrated as undifferentiated pleomorphic sarcoma, one resembled angiosarcoma, and there was still one case displaying bone and cartilage formation in the recurrent lesion after the IM therapy. In the previous studies, remarkable morphologic differences between the dedifferentiated components occurring *de novo* or after therapy have not been elucidated. However, we noticed a possible relationship between the rhabdomyosarcomatous GIST progression and the TKI therapy. Interestingly, all the above 10 cases with the rhabdomyoblastic differentiation had undergone long or short TKI treatment. Conversely, none of the *de novo* GISTs showed desmin expression. In one particular case with the shortest IM treatment for only 3 months, the expression of myogenic markers was weak and focal (13), compared with the strong and diffuse immunoreactivity observed in other cases after a long-term therapy (4, 6, 9, 11). We find it worthy of further discussion that the pressure of the TKI might have a certain effect on the phenotypic transformation and/or subclone selection of the GIST tumor cells.

Immunohistochemically, the dedifferentiated component completely lost or markedly decreased the expression of the CD117 and DOG1. The anaplastic area usually expressed P53 and showed an elevated ki-67 index. Therefore, the dedifferentiated GISTs often developed a diagnostic pitfall due to their unusual histological feature, confusing immunophenotype and rarity. Clinically, the GISTs may coexist with other malignancies. Thus, the dedifferentiated area, which juxtaposed with the classical GISTs, might be misinterpreted as carcinoma, rhabdomyosarcoma, or leiomyosarcoma (15) that collided with the GISTs. However, the molecular profile of the tumor might provide more valuable diagnostic information. If the driver mutations identified in the dedifferentiated area were consistent with those in the classic area, we could be more confident to infer the common origin of these lesions and draw the diagnosis of the dedifferentiated GIST.

Genetically, the mutation analysis revealed the dedifferentiation that could occur in the GISTs with the KIT mutation, PDGFRA mutation, or wild phenotype, which included the SDH-deficient subtype. The reported cases showed that the dedifferentiated GISTs, especially those after the therapy, were often accompanied by the deletion or mutation in the KIT exon 11, which we think might be due to the mutational frequency and prognostic significance of the driver gene alterations in the GISTs.

Notably, although the dedifferentiation after therapy correlates with the disease progression and treatment failure, it is quite different from the IM-resistant, which accounts for the majority of those events. Firstly, the dedifferentiation occurs after therapy or arises *de novo*, while the resistance only occurs under the pressure of TKI treatment. Secondly, the dedifferentiation and resistance might represent the different evolution of the cell clones. The dedifferentiation presents with the transdifferentiation into various histological patterns and loses immunopositivity for the CD117 and DOG1, while the resistance maintains the features of the GIST cells, both morphologically and phenotypically. In one reported case, the dedifferentiated nodules were observed against the background of the IM responsive tumor cell clones, indicating the existence of the distinct subclones within the tumor (8). Thirdly, the molecular mechanisms of the two were divergent. To our knowledge, the secondary KIT/PDGFRA mutations, which are the main reason for TKI resistance (16), rarely occur in the dedifferentiated GISTs (4). On the other hand, the genetic instability, such as loss of the heterozygosity (LOH) or the low-level KIT amplification, or the alternative escape mechanisms for the KIT-independent signaling pathways might play a role in the dedifferentiation (5). Previously, inactive mutations in the CDKN2A, TP53, and RB1 have been established as the secondary genetic events that correlated with the tumor progression and malignant behavior in the GISTs (17, 18). Recently, Du et al. found that the genetic alterations involving the RB1, SMARCB1, and MAX were the most common except the secondary KIT/PDGFRA mutations accounting for the IM resistance, whereas the P53 mutation was rarely involved (19). However, the strong and diffuse P53 protein expression and additional P53 gene mutation were reported in the dedifferentiated GISTs (3). In our case report, although the P53 mutation was not detected, the MDM2 amplification was displayed in the dedifferentiated component. The MDM2 proto-oncogene is a well-known inhibitor of the tumor suppressor P53. When amplified, the MDM2 facilitates the proteasomal degradation of the P53 and inhibits the P53-mediated transactivation. Hence, we speculated that the P53 and its associated genes, or other related signaling pathways, might affect the survival and proliferation of tumor cells, and play a role in the progression of the dedifferentiated GIST, as it has been demonstrated in the dedifferentiated liposarcoma, dedifferentiated solitary fibrous tumor (20), and dedifferentiated chordoma (21).

Among the 14 reported dedifferentiated GISTs with the available follow-ups, 3 patients died of the tumor dissemination. It seems that surgical resection or debulking might be the most effective option under the current existing treatment strategies. Few cases even had long time survival with the evidence of stable disease after surgery, and some even restarted with the TKI treatment based on other new lesions (9, 11, 13). Therefore, the dedifferentiation in these cases tended to be a local event instead of the systemic progress, although it transitioned into an aggressive morphology. In our case, the metastases to the regional lymph nodes were verified to be the conventional SDH-deficient GIST component, rather than the dedifferentiated cell group. Although it is consistent with the behavior of the SDH-deficient GISTs, the biologic behavior of the dedifferentiated GISTs remains to be elucidated and more cases are to be investigated.

In conclusion, we provided a case of the dedifferentiated SDH-deficient GIST in a young woman, which was accompanied by the MDM2 amplification in the dedifferentiated area. We summarized the clinicopathological features of the reported dedifferentiated GISTs and proposed that the P53 associated genetic changes might be responsible for the occurrence of dedifferentiation. Future studies to identify the mechanisms of dedifferentiation are required for a better understanding of the clinical significance of the dedifferentiated GISTs and optimize the individual treatment.

## Data availability statement

The original contributions presented in the study are included in the article/**Supplementary Materials**, further inquiries can be directed to the corresponding author.

## Ethics statement

Written informed consent was obtained from the participant/patient(s) for the publication of this case report.

## Author contributions

Q-XG made the diagnosis and wrote the manuscript. YD carried out the molecular experiments and analyzed the data. W-MZ performed immunohistochemical staining. J-WZ collected the clinicopathological materials. Z-HZ reviewed the slides and offered financial support. All authors contributed to the article and approved the submitted version.

## References

[1] WHO Classification of Tumors Editorial Board. WHO classification of tumours of soft tissue and bone. 5th ed. Lyon, France: International Agency for Research on Cancer (2020).

[2] Jung JH Im S Choi HJ Lee YS Jung ES. Gastrointestinal stromal tumor with dedifferentiation to undifferentiated pleomorphic sarcoma. Pathol Int (2013) 63(9):479–82. doi: 10.1111/pin.12095 24200161

[3] Antonescu CR Romeo S Zhang L Nafa K Hornick JL Nielsen GP. Dedifferentiation in gastrointestinal stromal tumor to an anaplastic KIT-negative phenotype: a diagnostic pitfall: morphologic and molecular characterization of 8 cases occurring either *de novo* or after imatinib therapy. Am J Surg Pathol (2013) 37(3):385–92. doi: 10.1097/PAS.0b013e31826c1761 PMC3728887 23348204

[4] Liegl B Hornick JL Antonescu CR Corless CL Fletcher CD. Rhabdomyosarcomatous differentiation in gastrointestinal stromal tumors after tyrosine kinase inhibitor therapy: a novel form of tumor progression. Am J Surg Pathol (2009) 33(2):218–26. doi: 10.1097/PAS.0b013e31817ec2e6 18830121

[5] Karakas C Christensen P Baek D Jung M Ro JY. Dedifferentiated gastrointestinal stromal tumor: Recent advances. Ann Diagn Pathol (2019) 39:118–24. doi: 10.1016/j.anndiagpath.2018.12.005 30661742

[6] Pauwels P Debiec-Rychter M Stul M De Wever I Van Oosterom AT Sciot R. Changing phenotype of gastrointestinal stromal tumours under imatinib mesylate treatment: a potential diagnostic pitfall. Histopathology (2005) 47(1):41–7. doi: 10.1111/j.1365-2559.2005.02179.x 15982322

[7] Bickenbach K Wilcox R Veerapong J Kindler HL Posner MC Noffsinger A. A review of resistance patterns and phenotypic changes in gastrointestinal stromal tumors following imatinib mesylate therapy. J Gastrointest Surg (2007) 11(6):758–66. doi: 10.1007/s11605-007-0150-y 17417711

[8] Vassos N Agaimy A Schlabrakowski A Hohenberger W Schneider-Stock R Croner RS. An unusual and potentially misleading phenotypic change in a primary gastrointestinal stromal tumour (GIST) under imatinib mesylate therapy. Virchows Arch (2011) 458(3):363–9. doi: 10.1007/s00428-010-1034-1 21191613

[9] Zheng S Huang K Jia J Li X Tao DY. Rhabdomyosarcomatous differentiation in gastrointestinal stromal tumors after imatinib resistance: a potential diagnostic pitfall. Exp Biol Med (Maywood) (2013) 238(1):120–4. doi: 10.1258/ebm.2012.012173 23479771

[10] Choi JJ Sinada-Bottros L Maker AV Weisenberg E. Dedifferentiated gastrointestinal stromal tumor arising *de novo* from the small intestine. Pathol Res Pract (2014) 210(4):264–6. doi: 10.1016/j.prp.2013.12.008 24484970

[11] Jiang X Anderson HB Guy CD Mosca PJ Riedel RF Cardona DM. Rhabdomyosarcomatous transformation of a gastrointestinal stromal tumor following treatment with imatinib. Case Rep Oncol Med (2015) 2015:317493. doi: 10.1155/2015/317493 25694839 PMC4324915

[12] Canzonieri V Gasparotto D Alessandrini L Miolo G Torrisi E Perin T. Morphologic shift associated with aberrant cytokeratin expression in a GIST patient after tyrosine kinase inhibitors therapy A case report with a brief review of the literature. Pathol Res Pract (2016) 212(1):63–7. doi: 10.1016/j.prp.2015.11.004 26616113

[13] Li L Khalili M Johannes G Baratam P Morano WF Styler M. Case report of rhabdomyosarcomatous transformation of a primary gastrointestinal stromal tumor (GIST). BMC Cancer (2019) 19(1):913. doi: 10.1186/s12885-019-6085-3 31514735 PMC6743131

[14] Malik F Santiago T Bahrami A Davis E McCarville B Newman S. Dedifferentiation in SDH-deficient gastrointestinal stromal tumor: A report with histologic, immunophenotypic, and molecular characterization. Pediatr Dev Pathol (2019) 22(5):492–8. doi: 10.1177/1093526619846222 31072206

[15] Insabato L Masone S Campione S Vigliar E Staibano S Tornillo L. Coexistence of primary gastric giant cell-rich leiomyosarcoma and gastrointestinal stromal tumor: report of a very rare combination and review of the literature. Int J Surg Pathol (2012) 20(1):74–8. doi: 10.1177/1066896911414018 21742646

[16] Liegl B Kepten I Le C Zhu M Demetri GD Heinrich MC. Heterogeneity of kinase inhibitor resistance mechanisms in GIST. J Pathol (2008) 216(1):64–74. doi: 10.1002/path.2382 18623623 PMC2693040

[17] Schneider-Stock R Boltze C Lasota J Miettinen M Peters B Pross M. High prognostic value of p16INK4 alterations in gastrointestinal stromal tumors. J Clin Oncol (2003) 21(9):1688–97. doi: 10.1200/JCO.2003.08.101 12721243

[18] Merten L Agaimy A Moskalev EA Giedl J Kayser C Geddert H. Inactivating mutations of RB1 and TP53 correlate with sarcomatous histomorphology and metastasis/recurrence in gastrointestinal stromal tumors. Am J Clin Pathol (2016) 146(6):718–26. doi: 10.1093/ajcp/aqw193 28028119

[19] Du J Wang S Wang R Wang SY Han Q Xu HT. Identifying secondary mutations in chinese patients with imatinib-resistant gastrointestinal stromal tumors (GISTs) by next generation sequencing (NGS). Pathol Oncol Res (2020) 26(1):91–100. doi: 10.1007/s12253-019-00770-6 31758409

[20] Akaike K Kurisaki-Arakawa A Hara K Suehara Y Takagi T Mitani K. Distinct clinicopathological features of NAB2-STAT6 fusion gene variants in solitary fibrous tumor with emphasis on the acquisition of highly Malignant potential. Hum Pathol (2015) 46(3):347–56. doi: 10.1016/j.humpath.2014.11.018 25582503

[21] Hung YP Diaz-Perez JA Cote GM Wejde J Schwab JH Nardi V. Dedifferentiated chordoma: clinicopathologic and molecular characteristics with integrative analysis. Am J Surg Pathol (2020) 44(9):1213–23. doi: 10.1097/PAS.0000000000001501 32427623

