# Supplementary material for: De novo dedifferentiated SDH-deficient gastrointestinal stromal tumor with MDM2 amplification: case report and literature review
Source: Front Oncol. 2023 Sep 14;13:1233561. doi: 10.3389/fonc.2023.1233561 (PMC10540086; doi:10.3389/fonc.2023.1233561)
Supplement: Supplementary file 1 [file Table_1.docx]

Supplementary Table 1: Clinicopathological characteristics of reported differentiated GISTs

| Reference no | Age/Gender | Location | Tumor size(cm) | Differentiated Morphology | Genotype | Treatment history |
| --- | --- | --- | --- | --- | --- | --- |
| 6 | 37/M | Stomach（Primary）；Intrabdominal（Recurrent / metastasis） | NA；6cm | Uniform and large epithelioid cells | KIT exon 11 deletion (W557_K558del) | secondary to imatinib treatment for 9 months |
| 6 | 44/M | Duodenum（Primary）; Liver(Metastasis) | 17cm;3.5cm | Tubulopapillar and epithelioid growth pattern | KIT exon 11 deletion (V569_L576del) | secondary to imatinib treatment for 24 months |
| 6 | 73/M | Rectum （Primary）; Liver(Metastasis) | 7.5cm;8.5cm | Epithelioid cells with rhabdomyoblastic differentiation | KIT exon 11 point mutation K558N | secondary to imatinib treatment for 14 months |
| 7 | 70/M | Stomach（Primary）, recurred 3 years later in original site | NA | Epithelioid differentiation and bone and cartilage formation | KIT exon 11 deletion (W557_K558del) | secondary to imatinib treatment for 24 months |
| 4 | 53/M | Small bowel（Primary）  Omentum&Liver(Metastasis) | 15cm | Rhabdomyoblastic differentiation | KIT exon 11 point mutation V559D | secondary to imatinib treatment for 54 months |
| 4 | 39/F | extensive metastasized when hospitalized | 6 to 20cm | Rhabdomyoblastic differentiation | KIT exon 11 deletion (Q556_T574del) | secondary to imatinib treatment for 14 months |
| 4 | 35/F | Stomach（Primary）, mesentery(Metastasis) | 11.3cm;3 to 7cm | Rhabdomyoblastic differentiation | KIT exon 11 point mutation V559D | secondary to imatinib treatment for 31 months |
| 4 | 57/M | gastrosplenic ligament（Primary）; Liver(Metastasis) | 9-16cm | Rhabdomyoblastic differentiation | KIT exon 11 deletion (Q556_T574del) | secondary to imatinib treatment for 33 months |
| 4 | 66/M | Stomach（Primary）, Peritoneum(Metastasis) | 20cm;3.5cm | Rhabdomyoblastic differentiation | PDGFRA exon 18 deletion, KIT wild-type | secondary to imatinib treatment for 20 months |
| 8 | 62/F | Stomach（Primary, diagnosed by core biopsy） | 13.3cm | Epithelioid and pleomorphic cell nodules with brisk mitotic activity observed in the background of the imatinib responsive hyalinized tumor | Wild type | secondary to imatinib treatment for 8 months |
| 2 | 51/M | Stomach（Primary） | 11cm | Undifferentiated pleomorphic sarcoma | KIT exon 11 deletion (V555_V559del) | de novo |
| 9 | 55/M | jejunal（Primary）, Liver(Metastasis) | 6.5cm; NA | Rhabdomyoblastic differentiation | KIT exon 11 point mutation V559D | secondary to imatinib treatment for 16 months |
| 3 | 23/M | Stomach（Primary）, Liver (Metastasis) | NA | CK+, desmin+ | Wild type | secondary to imatinib treatment |
| 3 | 40/F | Stomach（Primary）, Peritoneal (Metastasis) | 8 cm | CK+ | Wild type | de novo |
| 3 | 55/M | Stomach（Primary） | 18 cm | NA | KIT exon 11 deletion (W557_K558del) | de novo |
| 3 | 48/M | Stomach（Primary）, Peritoneal (Metastasis) | 5.5 cm | CK+ | Wild type | de novo |
| 3 | 58/M | Rectum（Primary） | 6 cm | NA | KIT exon 11 deletion (W557_K558del) | de novo |
| 3 | 53/M | Stomach（Primary）, Peritoneal (Metastasis) | 7 cm | CK+ | Wild type | de novo |
| 3 | 60/M | Small bowel（Primary）  Liver& Peritoneal (Metastasis) | 7.5 cm | Endothelial lineage differentiation | KIT exon 11 point mutation V559G  KIT exon 13 point mutation V654A | secondary to imatinib treatment for 40 months |
| 3 | 65/M | Colon（Primary）, Peritoneal (Metastasis) | 25 cm | NA | KIT exon 11 deletion (K550_W557del)  KIT exon 13 frameshift mutation (V643Sfs6*) | secondary to imatinib treatment for 48 months |
| 10 | 52/F | Small intestine（Primary） | 30cm | Anaplastic cells with an epithelioid morphology that were negative for AE1/3, desmin | Wild type | de novo |
| 11 | 47/M | Stomach（Primary）；pelvic cavity(Metastasis) | 10.4cm；5.5cm | Rhabdomyosarcomatous Transformation | KIT exon 11 deletion (W557_K558del) | secondary to imatinib treatment for 8 months |
| 12 | 52/M | Ileum（Primary） | 15cm | Epithelioid/ clear cell histology showing E-cadherin and Pankeratin positivity | KIT exon 11 point mutation (V560D) | secondary to TKIs (imatinib, sunitinib, nilotinib) treatment for 8 years |
| 13 | 75/M | Stomach（Primary） | 29cm | Rhabdomyosarcomatous Transformation | KIT exon 11 insertion- deletion (W557_V559delinsC) | secondary to imatinib treatment for 3months |
| 14 | 18/M | Stomach（Primary） | 23cm | Pleomorphic epithelioid cells | SDH-Deficient | de novo |
| our | 32/F | Stomach（Primary） | 10cm | SDHB- | SDH-Deficient | de novo |

NA: not available
